# From Cosmetic Abuse to Clinical Mismanagement: A National Simulated Patient Study Assessing Community Pharmacists’ Stewardship of Topical Corticosteroids in Jordan

**DOI:** 10.3390/pharmacy14010031

**Published:** 2026-02-09

**Authors:** Derar H. Abdel-Qader, Abdullah Albassam, Esra’ Taybeh, Nadia Al Mazrouei, Rana Ibrahim, Reham Aljalamdeh, Salim Hamadi, Alia Saleh, Sahar Jaradat, Shorouq Al-Omoush

**Affiliations:** 1 Faculty of Pharmacy & Medical Sciences, University of Petra, Amman 11196, Jordan; 2 Department of Pharmacy Practice, Faculty of Pharmacy, Kuwait University, Kuwait City 12037, Kuwait; albassam@hsc.edu.kw; 3 Department of Applied Pharmaceutical Sciences, School of Pharmacy, Isra University, Amman 11196, Jordan; esra.taybeh@iu.edu.jo; 4 Department of Pharmacy Practice and Pharmacotherapeutics, College of Pharmacy, University of Sharjah, Sharjah 27272, United Arab Emirates; nalmazrouei@sharjah.ac.ae (N.A.M.); ribrahim@sharjah.ac.ae (R.I.); 5 Faculty of Pharmacy, Philadelphia University, Amman 19392, Jordan

**Keywords:** adrenal cortex hormones, community pharmacy services, Jordan, patient safety, patient simulation, professional practice, skin lightening preparations

## Abstract

**Background:** Topical Corticosteroids (TCS) are potent therapeutic agents associated with severe local and systemic adverse effects if misused. In Jordan, the unauthorized supply of TCS for cosmetic purposes and the mismanagement of dermatological conditions remain significant public health concerns. This study utilized a repeated-measures simulated patient (SP) methodology to evaluate community pharmacists’ stewardship of TCS across a spectrum of clinical risks. **Methods:** A national cross-sectional study was conducted across 380 randomly selected community pharmacies in Jordan. Each pharmacy received four covert visits (N = 1520) corresponding to four distinct clinical scenarios representing different risk levels: cosmetic whitening, acne management, fungal infection, and pediatric diaper rash. The primary outcome was appropriate Practice, defined as the refusal to dispense unsafe medication or the provision of evidence-based alternatives. **Results:** Stewardship behavior varied significantly by clinical context (*p* < 0.001). Pharmacists demonstrated a hierarchy of safety, adhering to guidelines most strictly in the Pediatric scenario (82.1% appropriate refusal) but frequently abandoning safety standards in the Cosmetic scenario (30.0% appropriate refusal). Notably, 70.0% of pharmacists dispensed potent steroids for facial whitening, and 26.1% voluntarily offered to compound unauthorized steroid mixtures (*Khaltat*). In the acne scenario, 52.1% dispensed the contraindicated TCS, while only 37.9% appropriately switched the patient to an evidence-based alternative. In the fungal scenario, 60.0% failed to visually inspect the lesion, leading to a 40.0% rate of inappropriate TCS dispensing. Multivariable regression indicated that pharmacists working in chain pharmacies (aOR: 2.15, 95% CI: 1.68–2.75) and those holding advanced degrees (PharmD/MSc) (aOR > 1.38) were significantly more likely to practice appropriate TCS stewardship. High workload (>200 prescriptions/day) was a significant barrier to safety (aOR: 0.55). **Conclusions:** Community pharmacists in Jordan exhibited selective TCS stewardship, demonstrating high vigilance for pediatric safety, but widespread illegal practice regarding cosmetic misuse and differential diagnosis that may be unethical. The study results warrant the need for further urgent research to understand why these practices are occurring and how best to address them.

## 1. Introduction

Topical Corticosteroids (TCS) remain core to the management of numerous inflammatory dermatoses because of their marked anti-inflammatory, immunosuppressive, and antiproliferative actions [1]. However, their therapeutic index (benefit-to-risk profile) remains greatly dependent on potency, vehicle, and duration of application, as tailored to patient age and body site of application [2]. Though these remain highly effective when used properly, misuse, especially when applying high-potency preparations over thinner-skinned zones (the face and genital region), as well as when these remain occluded, provokes marked localized cutaneous hazards, which include cutaneous atrophy, telangiectasias, striae, and rosacea precipitated by steroid application [2,3]. There remain concerns regarding systemic absorption, particularly in infancy and childhood, and regarding iatrogenic precipitated Cushing’s Syndrome and hypothalamic and adrenal axis suppression [4].

Despite these established dangers, abuse of TCS is a developing concern within global public health circles, often resulting from ease of availability within a non-consultative setting [1]. Within Jordan itself, all Topical Corticosteroids (TCS) are classified as Prescription-Only Medicines (POM) by the Jordan Food and Drug Administration (JFDA). There are no TCS products legally available as over-the-counter (OTC) medications in Jordan [5]. Consequently, the supply of these medications without a valid medical prescription is illegal. It is within this permissive environment that community pharmacists emerge as critical gatekeepers of dermatological stewardship. This study therefore observes the prevalence of illegal supply in the first instance, followed by an assessment of the clinical appropriateness of the dispensing decision.

One of the disturbing aspects of such abuse, particularly prevalent in the Middle East and North African region, is the topical usage of corticosteroids for the purpose of skin whitening [6]. The need for lighter skin has resulted in the creation of an underground market where pharmacists are known to distribute strong steroids as well as formulate illicit compounds on their own known locally as ‘*Khaltat*’ (mixtures), which typically include combinations of potent steroids, hydroquinone, and retinoid. These do not just cause irreparable damage but further contribute to an addictive pattern owing to the rebound phenomenon once these compounds are stopped [7]. Furthermore, supplementary hydroquinone usage, even when used in these illegitimate compounds, leads to exogenous ochronosis, which is a pigmentary dermal problem [8,9].

Apart from cosmetic misuse, the misuse of these treatments in the management of some dermatologic conditions represents a considerable patient safety risk. In the case of acne vulgaris, the use of TCS would be contraindicated due to the risk of accelerating the infectious process and producing steroid-induced acne; however, these are often demanded by patients who seek rapid reduction in inflammation [10]. Also, the use of TCS without thorough examination in the case of suspected fungal dermatitis (tinea) represents a considerable risk because it will obscure the underlying dermatitis while the fungal organism multiplies, resulting in the condition of tinea incognito that slows the diagnosis and adequate management of the condition [11,12]. Lastly, the use of potent TCS in the case of diaper rash in children would pose a considerable risk in view of the considerable enhancement of skin permeability by the occlusive diapering area [13].

Community pharmacists have a pivotal role to play here, considering the fact that they often serve as the first healthcare professionals for patients to approach for skin problems. The global criteria of good pharmacy practice highlight patient evaluation and rational dispensing of medicines [14]. However, there is a dearth of existing literature on actual professional conduct.

This is the first national survey in Jordan to assess pharmacist behavior in four different risk-based scenarios: (1) Whitening misuse (Cosmetic), (2) Contraindicated use (Acne), (3) Risk of diagnostic error (Fungal/Tinea), and (4) Vulnerable high-risk populations (Pediatric). The aims were to measure irrational dispensing rates, analyze diagnostic questioning and counseling adequacy, and investigate predictors of appropriate use.

## 2. Materials and Methods

### 2.1. Study Design and Setting

This was a national cross-sectional study employing simulated patient (SP) methodology. This design was selected as the gold standard for objectively assessing community pharmacists’ stewardship of TCS using four standardized scenarios representing common misuse and mismanagement pathways: cosmetic whitening, acne request for potent steroids, suspected fungal infection misdiagnosed as allergy and infant diaper rash request for TCS. Simulated patient methods are widely used to evaluate actual dispensing and counseling behaviors in routine practice [15] and have been applied in Jordan to examine non-prescribed medicine supply [16]. The study was conducted in community pharmacies across different regions in Jordan.

This study was reported in accordance with the STROBE (Strengthening the Reporting of Observational Studies in Epidemiology) guidelines.

### 2.2. Study Population and Sampling

The target population of this study included all licensed community pharmacists who worked in patient-facing roles in community pharmacies across Jordan. All community pharmacists included in the final analysis provided informed consent via a deferred consent protocol (detailed in Section 2.5). Those working in non-patient-facing roles (e.g., administrative, academic, or industry roles), pharmacy students, or internships, or those that declined to provide informed consent, were excluded. The stratified proportionate random sampling method was applied to ensure national sampling representation based on pharmacies’ geographical distribution. Based on the number of pharmacies in each region, the population was subdivided into three groups as follows: Central Region (70.4%), North Region (23.9%), and the South Region (5.6%).

The sampling frame was the official registry of licensed community pharmacies provided by the Jordan Pharmacists Association (JPA) (N = 5048, [17]). Using the Raosoft online sample size calculator (Raosoft, Inc., Seattle, WA, USA), we determined our minimum required sample size (n = 380) based on a 5% margin of error, a 95% confidence interval, and a conservative 50% response distribution to achieve the greatest variance in the sample. The sample sought was proportionately split among the strata to get 268 in the Central Region, 91 in the North Region, and 21 in the South Region. The process of selecting the study subjects entailed randomly sampling the requisite numbers of pharmacies from the lists that had been compiled for each of the strata utilizing a computer-based random number generator. Then, a subsequent random sampling was done for one practicing pharmacist from each one of these pharmacies.

### 2.3. Data Collection Instrument and Validation

Data collection was undertaken using a structured observation checklist (questionnaire) which was designed using a detailed literature review related to evidence on TCS potency-related risks and counseling requirements [1,2,11,18], pediatric systemic risk and HPA-axis suppression warnings [4,13,19,20], skin-lightening harms related to corticosteroids and hydroquinone [6,7,8,9,21,22], and evidence on tinea diagnosis and tinea incognito caused by steroid misuse [10,12,23]. Other sections’ questions were based on other studies [24,25]. The checklist was hosted on a secure mobile data collection platform (Google Forms) to allow SPs to record data immediately upon exiting the pharmacy, thereby minimizing recall bias. The questionnaire was intended to be comprehensive and was divided into four sections as follows:

- **Section A: Pharmacy and Visit Demographics:** Collected observable data on the visit scenario, pharmacist’s gender, years of experience, highest education and prior training in dermatology/steroid safety, as well as pharmacy characteristics, such as type (independent vs. chain), region (North, South and Central), and an estimate of workload at the time of the visit.
- **Section B: History Taking and Assessment:** Evaluated the pharmacist’s adherence to basic clinical assessment questions and safety information-gathering process using a checklist of key questions regarding presence of allergy or pregnancy/breastfeeding. The basic clinical assessment questions aid to help the pharmacist consider the patient’s choice when managing requests for over-the-counter medicines for managing minor ailments and explicitly encourages the pharmacist to ask, Who is this patient?, What are the symptoms?, How long have the symptoms been there?, Action already taken? and Medication currently taking?
- **Section C: Scenario-Specific Clinical Competency:** Measured the dispensing decision. It was tailored to each of the four scenarios to capture specific clinical competencies:
  ○ Scenario 1: Assessed whether the pharmacist validated the cosmetic misuse of steroids or offered to compound a mixture (Khaltat).
  ○ Scenario 2: Assessed knowledge of contraindications (i.e., avoiding steroids for acne vulgaris).
  ○ Scenario 3: Assessed diagnostic inquiry (visual inspection of the lesion) and the appropriateness of the product selected (antifungal vs. corticosteroid).
  ○ Scenario 4: Assessed adherence to safety contraindications regarding the use of potent TCS in infants.
- **Section D: Counseling and Safety Information:** Evaluated the quality of the verbal counseling provided if a product was dispensed. It measured the presence of four key information elements: frequency of application, duration of use, appropriate area of application, and warning regarding potential local or systemic side effects (e.g., skin atrophy, systemic absorption).

After this, the scenarios were thoroughly content validity tested by a multidisciplinary committee consisting of three experts: a specialist dermatologist, a senior community pharmacist, and a professor of clinical pharmacotherapy. It was ensured that all scenarios were clinically valid and appropriate for the Jordanian setting. Small changes were later made to meet the suggestions of the committee. Subsequently, a pilot test was done to assess a total of 8 SP visits to the pharmacy, with the objectives to: (1) validate the realism and clarity of the scenarios, (2) validate the data collection form to be complete and correct for precise recording in an effective and adequate manner by the SPs and (3) modify the training protocol for the SPs.

The reliability of data collection was ensured by the rigorous standardization of SP performance achieved through an intensive training procedure conducted by the principal investigator (professor of clinical pharmacotherapy) and a specialist dermatologist. The use of an extremely formalized check-list type of form helped reduce subjective data interpretation, as all data collection was consistent across all visits.

### 2.4. Simulated Patient Scenarios, Recruitment and Training

Four cases for SPs have been devised based on a review of the existing global clinical guidance on skin conditions and the use of TCS.

- **Scenario 1, Cosmetic Whitening:** the SP presented the complaint of a female patient seeking a particular strong corticosteroid, for example, clobetasol or another strong formulation, to use in whitening the dark spots of the face. The expected target response was to not give out the TCS, while advising the patient of potential dangers, such as atrophy of the skin and steroid rosacea, among others.
- **Scenario 2, Acne Management:** The SP acted as a sibling of a patient, presenting a high-resolution photograph on a smartphone showing a young adult with mild-to-moderate inflammatory facial acne and requested a potent TCS for quick fix prior to an event. The most desirable response for this SP would be non-dispensation of the TCS and the provision of advice regarding evidence-based over-the-counter alternatives (e.g., Adapalene or benzoyl peroxide).
- **Scenario 3: Suspected Fungal Infection:** The SP acted as a sibling, presenting a smartphone photograph of a lesion on the arm characterized as a pruritic reddish expanding ring (classic tinea corporis) and was erroneously attributed to an allergy and a request for a TCS. The desired optimal response would involve the lesion being assessed and a topical antifungal medication being advised instead of a steroid to prevent tinea incognito.
- **Scenario 4, Pediatric Diaper Rash:** The role of the SP presented as the parent showing a photograph of a 6-month-old infant medication for severe diaper rash, seeking specifically a cream in a green tube (Dermovate^®^, clobetasol). The best expected response would be a refusal of the medication and referral to a pediatrician, because clobetasol is a Class I steroid, posing substantial risks of skin thinning, increased infection susceptibility, systemic absorption, and potential hormone suppression.

The sample size of 380 community pharmacists and representation across three regions of Jordan required engagement of sixteen research assistants to act as SPs (eight for the Central Region, four for the North, and four for the southern part of Jordan). The pool of SPs included a variety of ages and both female and male subjects, in order to suit specific scenarios (e.g., female for cosmetic/maternity). All SPs were trained in a one-day training session with a focus on standardization of scenarios, role-playing, and entering data accurately. Also, inter-rater reliability was determined before the data was collected. All data collectors had a Cohen’s kappa coefficient above 0.80 in the video scores.

### 2.5. Data Collection Procedure and Post Hoc Consent

The data collection was conducted over a 12-week period from September to November 2025. The SP randomly approached one of the on-duty community pharmacists per pharmacy chosen. The targeted community pharmacist per pharmacy was consistent for each of the four interactions. To prevent anomalies, the first SP took note of the physical characteristics and time of pharmacist shift, and subsequent interactions were arranged according to these factors, with a minimum of 2 days separating interactions, to avoid arousing suspicion. In cases where targeted community pharmacists were not accessible, SPs immediately withdrew without executing the scenario and revisited later.

To ensure the observation and the characteristics of the pharmacists were not exploitative and that the initial undercover observation was not undermined, the data were collected in two stages for each pharmacy site visit:

- **Step 1, Repeated Covert SP Interactions (Visits 1, 2, 3, and 4):** Every pharmacy underwent four separate visits based on the four scenarios studied. On completing every visit, the assigned SP would enter the pharmacy and act out the given scenario discretely. The major function of the SP remained the execution of the given script and noting the clinical behavior and dispensing decision taken by the pharmacist. As soon as the interaction ended, the SP would enter the details on the electronic data form. There would be no debriefing process post visits 1, 2, and 3 to maintain the blinding process for the next scenarios.
- **Step 2, Immediate Revisit, Debriefing, and Cumulative Verbal Consent (After Visit 4):** The SP immediately, no later than 5 min from the completion of the fourth interaction, went back to the same pharmacy and met the pharmacist whom they had just finished observing. A debriefing process was undertaken. The researcher immediately, right after the fourth interaction, made known the purpose of the study and what had transpired between the first and fourth interactions with the pharmacist under observation. The cumulative data from all four interactions could then be used once the pharmacist gave their consent, which had to be given verbally, or else none of the data could be used from the particular pharmacy. The consent status and demographic information were recorded on the final electronic form and then connected to the last three visits through a pharmacy ID code.

Data collection was conducted on a continuous basis until the requisite sample size was achieved.

### 2.6. Outcome Measures and Variables

- Primary Outcome: The primary outcome was appropriate practice, a binary variable (appropriate vs. inappropriate) determined by the pharmacist’s adherence to the expected optimal outcome mentioned before for each clinical scenario. Specifically, a visit was coded as appropriate if it meets the expected outcome. Appropriate practice was operationally defined as the refusal to dispense unsafe or contraindicated medications, paired with the provision of evidence-based alternatives or medical referral as follows: scenario 1: Refusal to dispense the TCS; scenario 2: Refusal to dispense + Assessment + Advice to use exfoliants/Benzoyl peroxide; scenario 3: Refusal to dispense + Assessment + Advice to use an antifungal; and scenario 4: Refusal to dispense + Referral to a pediatrician. Conversely, inappropriate practice was operationally defined as any interaction where the pharmacist failed to uphold these safety standards or engaged in irrational dispensing. This included the direct supply of the requested corticosteroid for cosmetic whitening or the unauthorized offer to compound steroid mixtures (*Khaltat*). In the acne and fungal scenarios, practice was deemed inappropriate if the pharmacist dispensed contraindicated corticosteroids, provided combination creams without performing a visual assessment, or failed to suggest evidence-based alternatives. In the pediatric scenario, any instance where a Class I steroid was dispensed for an infant or where the pharmacist failed to warn of the risks of systemic absorption and hormone suppression was categorized as inappropriate.
- Secondary Outcomes: These included:
  ○ The quality of history taking, measured by the number of basic clinical assessment questions and safety questions asked.
  ○ The rate of irrational dispensing, defined as the percentage of encounters where a TCS was dispensed (without a prescription) inappropriately.
  ○ The provision of specific safety information (frequency, duration, area, side effects) was measured to assess whether pharmacists attempted to mitigate harm (e.g., warning against large surface area application) in cases where they chose to dispense the medication.
- Independent Variables: Predictor variables included pharmacist’s gender, years of experience, educational degree, training courses, pharmacy type, pharmacy location, and workload at the time of the visit.

### 2.7. Ethical Considerations

The protocol of the study was completely approved by the Institutional Research Board (IRB) of the University of Petra (**S/25/8/2025**). As behavioral observation in the study would alter the actions (Hawthorne effect) of the pharmacist and thus affect the outcome of the findings, the IRB granted a waiver of prior informed consent. Instead, a deferred consent (post hoc) mechanism was utilized. Pharmacists were not aware they were being observed during the interactions. However, immediately following the final visit, the pharmacists were debriefed, the study was explained, and their individual consent was sought to use the recorded data. Pharmacists had the right to refuse, in which case their data was immediately destroyed. Only data from pharmacists who provided this explicit post hoc consent were retained for analysis.

Moreover, in consideration of the privacy of the participants and pharmacies involved in the study, the names of the community pharmacists and the pharmacies were not recorded. All the data collected in the study were anonymous before undergoing analysis.

### 2.8. Statistical Analysis

All information was coded, cleaned, and analyzed by the computer program SPSS, version 28.0, by IBM Corp., Armonk, NY, USA. Descriptive statistical analysis, such as frequencies and percentages, was used on demographics and outcome measures.

Inferential statistics were employed to analyze the data. Pearson’s Chi-square test (or Fisher’s exact test where cell counts were low) was used to assess associations between categorical independent variables and the primary outcome. To determine differences in the level of TCS stewardship practices, such as rates of dispensing and counseling rates, for the four clinical scenarios, Cochran’s Q test was employed. To identify differences for those variables that returned significant results in the overall test, McNemar’s test was used to perform post hoc comparisons after applying the Bonferroni adjustment for multiple comparisons.

In order to determine the independent predictors of the primary outcome, a multivariable logistic regression model was constructed. Univariate analyses were first performed; variables with *p* < 0.25 were considered for inclusion in the final model. Multicollinearity among independent variables was assessed using a correlation matrix and Variance Inflation Factor (VIF), with a threshold of VIF < 0.05 indicating no significant multicollinearity. The goodness-of-fit of the final model was evaluated using the Hosmer–Lemeshow test, and the amount of variance explained was estimated using Nagelkerke R2. Results are presented as Crude Odds Ratios (cOR) and Adjusted Odds Ratios (aOR) with 95% Confidence Intervals (CI). A *p*-value of ≤0.05 was considered statistically significant.

## 3. Results

### 3.1. Demographic Characteristics

A total of 412 community pharmacies were randomly approached during the study period. Following the immediate debriefing and request for post hoc consent, 32 pharmacists (7.8%) declined to participate or withheld consent for the use of their data. These encounters were immediately excluded and destroyed in accordance with the ethical protocol. Consequently, the final analysis included 1520 simulated patient visits across 380 participating community pharmacies (four visits per pharmacy). The demographic characteristics of the participating pharmacists are detailed in Table 1. The majority of pharmacists were female (62.1%) and held a Bachelor of Pharmacy degree (70.0%). Approximately one-third of the visits (35.0%) were conducted in chain pharmacies.

### 3.2. Comparison of Assessment and Dispensing Practices Across Scenarios

There was a statistically significant difference in clinical performance across the four scenarios (*p* < 0.001) (Table 2). In terms of history-taking, the pharmacists were more likely to ask the question of the patient’s identity (Who is the patient?) in the pediatric diaper rash case (75.0%) than the cosmetic whitening case (30.0%). Nevertheless, full safety questioning was rare, with questions on allergy or pregnancy/breastfeeding considered in fewer than 6% of all encounters in the three situations.

The overall dispensing behavior was very diverse when considering clinical indication. The highest irrational dispensing of the demanded TCS was recorded in the cosmetic whitening indication (70.0%), followed by acne management indication (52.1%). On the other hand, pharmacists adhered most to safety principles in the pediatrics indication and refused to dispense the potent TCS in 82.1% of cases.

There were also differences in the quality of counseling (*p* < 0.001). Although the advice on frequency was very high (>80%) in all groups, the most important advice on the area of application was given more frequently in the pediatric setting (74.6%) than in the cosmetic setting (28.6%).

### 3.3. Scenario-Specific Clinical Competency

Table 3 illustrates the gaps in clinical knowledge. For Scenario 1, 65.0% of pharmacists mistakenly legitimized the patient’s perception that TCS are efficacious as whitening agents. Moreover, it was evident that 26.1% of pharmacists independently suggested preparing the steroid as a mixture (*Khaltat*) to be taken by the patient. For Scenario 2, only 28.9% of pharmacists correctly stated that TCS are not indicated in acne. Nevertheless, 37.9% of pharmacists referred the patient correctly to evidence-based treatment. For Scenario 3, there was no proper diagnostic inquiry by the pharmacists. Only 40.0% asked about the morphology of the lesion, and 40.0% prescribed pure TCS, which might lead to tinea incognito. In Scenario 4, the pharmacists demonstrated profound knowledge about safety. Indeed, 75.0% re-verified the age of the infant, and 42.1% correctly indicated potential systemic absorption.

### 3.4. Predictors of Appropriate Stewardship

Multivariable logistic regression analysis (Table 4) identified several independent predictors of appropriate practice. Assessment of multicollinearity confirmed that predictor variables were independent, with no significant cross-correlations observed (Supplementary Table S1). The final model demonstrated adequate fit (Hosmer–Lemeshow test: *p* = 0.391) and explained approximately 31.2% of the variance (Nagelkerke R^2^ = 0.312). Pharmacists working in chain pharmacies were more than twice as likely to adhere to stewardship guidelines compared to those in independent pharmacies (aOR: 2.15; 95% CI: 1.68–2.75, *p* < 0.001). Higher educational attainment was also associated with better outcomes; pharmacists holding a PharmD (aOR: 1.38) or MSc (aOR: 2.10) were significantly more likely to demonstrate appropriate practice compared to BSc holders. Conversely, high workload was a significant barrier, reducing the odds of appropriate practice by 45% (aOR: 0.55, *p* = 0.001). Pharmacist gender and geographical region were not statistically significant predictors.

## 4. Discussion

As far as we are aware, this is the first nationwide study conducted within Jordan that made use of a multi-scenario simulated patient study design aimed at the assessment of community pharmacists’ use and distribution of TCS based on various clinical risk scenarios. Interestingly, results indicate a significant disparity in professional behavior: a safety ranking within which the evaluated community pharmacists showed high levels of adherence with safe guidelines among vulnerable patients (infants) but showed lower adherence regarding requests posed within the context of cosmetics/lifestyle scenarios. This was clearly indicated by the fact that, whereas over 80% showed appropriate denial of the distribution of potent TCS among infants, 70% proceeded with the distribution of the same high-risk drug among patients requesting its use as a means of beautification through facial whitening.

The most noticeable outcome of this research was the variation in practice, suggesting a pattern of decision-making where adherence with guidance from the theoretical practice of stewardship (dispensing regulations) appears to vary significantly depending on the clinical context and patient demographic presented. In Scenario 4, 82.1% of pharmacists were correct in refusing the potent TCS to the infant, while in Scenario 1, the refusal rate decreased to 30.0% in the case of the cosmetic whitening products. Such discrepancies show that the pharmacists in Jordan demonstrated the capacity to apply safety guidelines in pediatric cases regarding TCS supply, as obviously exemplified by their treatment of infants, yet dispensed the medication despite the existence of strict regulatory guidelines prohibiting such supply. While the specific motivations were not qualitatively explored in this study, this behavior occurs within a private sector environment where patient satisfaction and business viability are often competing pressures. Such a pattern of illegal stewardship follows previous research in the pharmacological use of antibiotics, where societal demand managed to override the professional advice [16]. Thus, further research is required in this context to determine whether business pressures or other external factors were influencing decision-making or not.

While the cosmetic condition portrayed unauthorized supply, the acne condition contradicts international guidelines too. Against the background of international guidelines clearly stating that TCS should not be dispensed to patients for acne vulgaris [10], 52.1% of pharmacists dispensed the requested medications. This indicates that many pharmacists provided a treatment that may temporarily mask apparent symptoms (redness and inflammation) without addressing the underlying condition (bacterial growth and hyperkeratosis of the follicle).

The clinical risk involves the medicine providing the patient with an immediate aesthetic advantage through erythema blanching while simultaneously inhibiting local cutaneous immunity, hence promoting growth of *Cutibacterium acnes* bacteria [3]. Worth noting is the fact that from the data presented above, the pharmacist did not reveal the underlying mechanism of transmission of information to the patient, as evidenced by 22.1% of those reporting the risk of rebound or the risk of worsening the infection. On the other hand, the scenario also brought to light an opportunity that exists regarding suitable community care. About 38% of pharmacists appropriately refused the use of TCS and advised the patient to seek an alternative over-the-counter medication (adapalene and benzoyl peroxide). There was thus the possibility of therapy substitution that existed within the health care system. It will be important to promote such health care, from simple gatekeeping to advice on health care.

As far as the case of the fungal infection (Scenario 3) is concerned, a significant deficiency in handling the suspected case of the fungal infection existed. Indeed, in the case of an undifferentiated erythematous and annular rash, the assessment of the rash for the lack of infectious causes has to be done before anti-inflammatory therapy [12]. Conversely, it was found that 60% of pharmacists did not examine the rash and its appearance and dispensed without visual inspection based on the subjective complaint of itch in the presence of infection. The failure to assess here significantly increased the risk of therapeutic failure, and of the pharmacists, 60% prescribed a drug that contained a corticosteroid (whether alone or in combination) for what likely was a dermophytic infection. This is the fundamental source of tinea incognito, a condition of masking clinical signs, whereby the corticosteroid inhibits the immune system within the area and prevents inflammation from being apparent, thus facilitating the deeper invasion of the fungus into the dermis [11,23]. Combination creams (steroids and antifungals), which were observed in 20% of encounters, hold particular concerns in terms of their extensive use by the public as observed in these encounters. Even if perceived by healthcare practitioners, particularly those in pharmacies, as safe or broad-spectrum therapy in ambiguous dermatitis, these combination medications can affect dermatologic presentation and lead to treatment failures or reoccurrences due to infections. The role of the community pharmacist in Jordan is already established as that of a dispenser of therapy rather than an evaluator of pathology, but this paradigm must change if iatrogenic dermatologic disease is to be prevented. Contrary to the results seen within adult scenarios, the pediatric diaper rash case scenario (Scenario 4) showed the highest level of adherence to regulations, achieving an 82.1% refusal rate on potent TCS. It indicated that legal practices were significantly higher in infant patients compared to cosmetic or lifestyle-related scenarios.

While generic advice (e.g., frequency of use) was high across all scenarios, the specific safety warning regarding the area of application varied drastically. It was significantly higher in the pediatric scenario (74.6%) compared to the cosmetic scenario (28.6%). This disparity highlights a variation in practice; even among those who dispensed the medication, pharmacists provided specific safety warnings regarding the area of application more frequently in the pediatric scenario compared to the cosmetic interaction. In this respect, the diaper area represents a full-thickness occlusive dressing, increasing systemic corticosteroid bioavailability up to 100 times, posing an acute risk of iatrogenic Cushing’s syndrome and HPA axis suppression [13].

Nevertheless, it is important to point out that 17.9% of pharmacists also dispensed clobetasol to a 6-month-old infant. Even though this was in the minority, this was a dangerous act of violating safety. Class I steroids (super potent agents like clobetasol) for an infant’s intertriginous space are not only contraindicated practice but represent a significant deviation from safety standards. This finding highlights that despite higher general adherence in the pediatric setting, a subset of pharmacists still dispensed contraindicated high-potency steroids to infants, representing a significant safety concern.

Multivariate regression analysis provided vital information regarding non-clinical predictors in relation to pharmacy practice. The strongest predictor of responsible practices remained practice setting, where pharmacists working in chain pharmacies were over twice as likely (aOR = 2.15) to practice according to stewardship guidelines in comparison to independent pharmacies. The reasons for this significant difference in practice between chain and independent pharmacies were not evaluated in this study. Notably, this difference may be due to the safeguard offered by corporate management structure. Further research is warranted to understand whether organizational structures, such as auditing systems or remuneration models, influence these observed differences in stewardship.

Moreover, this validation provides support for the effectiveness of advanced clinical education as well. Pharmacists with either a PharmD or postgraduate education perform their job significantly better than those with conventional education such as a BSc (aOR > 1.38). Notably, this is in line with the strategic change in Jordanian pharmacies over the past ten years, which changed their curriculum emphasis progressively from basic chemistry to patient care and clinical practices. It seems that the advanced education in therapeutics, as in PharmD, qualifies pharmacists and gives them confidence in dealing with patient demands and making differentials, such as dermatitis and fungus infections.

On the contrary, high workload was shown to be a major structural issue in bringing about a reduction in probability for appropriate practice by 45% (aOR: 0.55). This suggests that time constraints in high-volume settings may structurally impede the pharmacist’s ability to perform necessary clinical assessments.

The first strength of our work is its methodology. Through the utilization of the SP methodology, we were able to minimize the issue of social desirability bias, common to traditional Knowledge, Attitude, and Practice (KAP) studies on knowledge and attitudes of practitioners towards the safety of TCS, which have shown remarkably high theoretical knowledge on the subject by participants from Jordan [21]. Contrary to such findings, our observation study clearly indicated both a discrepancy between competence and performance, between what is known and what is actually done, and, through the utilization of our special study design, the fact that pharmacist stewardship is context dependent: the same pharmacist could act appropriately towards the infant and be less rigorous towards the cosmetic customer.

Despite this, there were some limitations that have to be noted to put the findings into perspective. First, although the best efforts were made to avoid detection, it is impossible to exclude the role of the Hawthorne effect completely. It is likely that the pharmacist, having some idea that the encounter might be a simulation, perhaps towards the end of the rotation, might have behaved differently, towards demonstrating greater compliance, which might have led to an underestimate of the prevalence of malpractice. Second, the findings related to practice in community pharmacies only, and the practice patterns, particularly in outpatient pharmacies found in hospitals, where the influence of the commercial environment is not present, may be quite different. Third, because the encounter was observational and covert, the motivation that the pharmacists had for the choices that they made cannot be explored satisfactorily. It is important to distinguish that while the supply of TCS without a prescription is illegal in Jordan, the motivation may not always be unethical. In some instances, pharmacists may believe they are acting in the patient’s best interest by providing accessible treatment, thereby fulfilling a perceived professional duty despite the regulatory violation. Fourth, as this was a quantitative observation study, and we could not determine the internal motivations of the pharmacists (e.g., profit, lack of confidence, or desire to please the patient). Future qualitative research is required to understand the ‘why’ behind these behaviors. Finally, regarding generalizability, these findings likely reflect a broader culture of non-prescription supply in Jordan involving non-controlled POM. However, these behaviors may not generalize to strictly controlled substances (narcotics and benzodiazepines), which are subject to rigorous auditing and tracking systems not applied to dermatological products.

## 5. Conclusions

This national study revealed that adherence to stewardship guidelines among community pharmacists in Jordan varied significantly by clinical context. While the profession demonstrates high adherence in protecting infants from the risks of systemic absorption—evidenced by an 82% refusal rate in the pediatric scenario—refusal rates were significantly lower for cosmetic skin lightening. The finding that over a quarter of pharmacists actively facilitated the use of unauthorized steroid mixtures (*Khaltat*) indicates a prevalence of informal prescribing.

Furthermore, the study highlighted critical gaps in diagnostic competence, particularly the blind dispensing of TCS for fungal and acneiform conditions, which directly predisposed patients to iatrogenic harm, such as tinea incognito and steroid acne. Structural factors played a decisive role; the optimal performance of pharmacists in chain settings and those with advanced clinical degrees (PharmD) suggests that corporate governance and clinical education are protective against malpractice. Conversely, high workload served as a systemic barrier to effective history-taking. While this study was not designed to measure behavioral drivers, the Theory of Reasoned Action could provide a useful framework for future qualitative research. It would be valuable to investigate whether subjective norms regarding customer service in the private sector influence the gap between clinical knowledge and practice.

Future research must prioritize qualitative methodologies to explore the cognitive and environmental drivers of these practices. Specifically, understanding the barriers to and enablers of appropriate stewardship, whether stemming from commercial pressure, conflict avoidance, or gaps in clinical confidence, is essential for designing effective interventions that go beyond simple regulatory enforcement.

## Figures and Tables

**Table 1:** Demographic and professional characteristics of participating community pharmacists and pharmacies (N = 380).

Characteristic	Frequency (*N*)	Percentage (%)
Pharmacy Type		
Independent	247	65.0
Chain	133	35.0
Geographical Region		
Central	268	70.4
North	91	23.9
South	21	5.6
Pharmacist Gender		
Male	144	37.9
Female	236	62.1
Professional Experience		
<2 years	95	25.0
2–10 years	209	55.0
>10 years	76	20.0
Highest Educational Degree		
Bachelor of Pharmacy (BSc)	266	70.0
Doctor of Pharmacy (PharmD)	95	25.0
Master of Science (MSc)	19	5.0
Prior Training on Topical Corticosteroids		
Yes	114	30.0
No	266	70.0
Estimated Workload (Prescriptions/Day)		
Low (<50 prescriptions/day)	76	20.0
Medium (50–100 prescriptions/day)	190	50.0
High (>100 prescriptions/day)	114	30.0

**Table 2:** Comparison of pharmacist assessment, dispensing practices, and counseling across four clinical scenarios (N = 380 visits per scenario).

Variable	S1: Cosmetic (n = 380) N (%)	S2: Acne (n = 380) N (%)	S3: Fungus (n = 380) N (%)	S4: Pediatric (n = 380) N (%)	*p*-Value *
History Taking & Assessment					
Asked: Who is the patient?	114 (30.0) ^a^	133 (35.0) ^a^	152 (40.0) ^b^	285 (75.0) ^c^	<0.001
Asked: What are the symptoms?	95 (25.0) ^a^	114 (30.0) ^a^	209 (55.0) ^b^	228 (60.0) ^b^	<0.001
Asked: How long (Duration)?	38 (10.0) ^a^	57 (15.0) ^b^	76 (20.0) ^c^	114 (30.0) ^d^	<0.001
Asked: Action taken (Previous meds)?	23 (6.1) ^a^	38 (10.0) ^b^	45 (11.8) ^b^	57 (15.0) ^b^	0.002
Asked: Other Medication conditions?	11 (2.9) ^a^	15 (3.9) ^a^	19 (5.0) ^a^	22 (5.8) ^a^	0.210
Performed Safety Check (Allergies/Pregnancy/Breastfeeding)	15 (3.9) ^a^	22 (5.8) ^a^	19 (5.0) ^a^	11 (2.9) ^a^	0.152
Dispensing Outcome					
Dispensed Requested Drug	266 (70.0) ^d^	198 (52.1) ^c^	152 (40.0) ^b^	68 (17.9) ^a^	<0.001
Dispensed Alternative	42 (11.1) ^a^	72 (18.9) ^b^	114 (30.0) ^c^	133 (35.0) ^c^	<0.001
Refused & Referred	19 (5.0) ^a^	38 (10.0) ^b^	57 (15.0) ^c^	114 (30.0) ^d^	<0.001
Refused & Ended	53 (13.9) ^a^	72 (18.9) ^a^	57 (15.0) ^a^	65 (17.1) ^a^	0.184
Provision of Safety Information (Among those who dispensed)	(n = 308)	(n = 270)	(n = 266)	(n = 201)	
Explained Frequency	262 (85.1) ^a^	229 (84.8) ^a^	213 (80.1) ^a^	171 (85.1) ^a^	0.321
Explained Duration of Use	62 (20.1) ^a^	54 (20.0) ^a^	53 (19.9) ^a^	90 (44.8) ^b^	<0.001
Explained Area of Application	88 (28.6) ^a^	110 (40.7) ^b^	140 (52.6) ^c^	150 (74.6) ^d^	<0.001
Warned of Side Effects	31 (10.1) ^a^	41 (15.2) ^a^	27 (10.2) ^a^	80 (39.8) ^b^	<0.001
Primary Outcome					
Appropriate Practice **	114 (30.0) ^a^	182 (47.9) ^b^	190 (50.0) ^b^	312 (82.1) ^c^	<0.001

* *p*-values for categorical variables calculated using Pearson’s Chi-square test. Values in the same row not sharing the same superscript letter (a, b, c, d) are significantly different (*p* < 0.05) based on Bonferroni-corrected post hoc *Z*-tests. ** Appropriate Practice defined as: S1 (Refusal), S2 (Refusal + Assessment + Advice exfoliants/Benzoyl peroxide), S3 (Refusal + Assessment + Antifungal), S4 (Refusal + Referral).

**Table 3:** Pharmacists’ responses to scenario-specific clinical competency questions (N = 380 visits per scenario).

Questionnaire Item	Response (Yes)	%
Scenario 1: Cosmetic Whitening		
Did the pharmacist agree that TCS helps with whitening?	247	65.0
Did they warn about skin atrophy (thinning) or telangiectasia (visible veins)?	57	15.0 *
Did they offer to mix the steroid with other creams (making *Khaltat*)?	99	26.1
Did they recommend a safe cosmetic alternative (e.g., Vitamin C, natural compounds, etc.)?	95	25.0 *
Scenario 2: Acne Management		
Did the pharmacist explicitly state that TCS are bad/contraindicated for acne?	110	28.9 *
Did they explain that TCS might reduce redness but will cause rebound Steroid Acne?	84	22.1 *
Did they switch the recommendation to an evidence-based acne product (e.g., Benzoyl Peroxide/Adapalene)?	144	37.9 *
Scenario 3: Fungal Infection		
Did the pharmacist ask to see the lesion or ask for a specific description (e.g., Is it a ring?)?	152	40.0 *
Did the pharmacist mention the possibility of a Fungal Infection (*Tinea*)?	150	39.5 *
What was the primary recommendation?		
*Antifungal*	152	40.0 *
*Corticosteroid*	152	40.0
*Combination*	76	20.0
Scenario 4: Pediatric Diaper Rash		
Did the pharmacist re-confirm the baby’s age (6 months)?	285	75.0 *
Did they warn that potent TCS (clobetasol) can cause systemic absorption/hormonal issues in infants?	160	42.1 *
Did they strictly refuse to sell clobetasol for a baby?	312	82.1 *

* Indicates the proportion of pharmacists performing the correct clinical practice.

**Table 4:** Multivariable logistic regression analysis of factors associated with appropriate stewardship practice (N = 380 visits per scenario).

Variable	Appropriate Practice (N/Total) (%)	Crude OR (95% CI)	Adjusted OR (95% CI) *	*p* -Value
**Pharmacy Type**				
Independent (Ref)	395/988 (40.0%)	1.00	1.00	–
Chain	338/532 (63.5%)	2.61 (2.09–3.26)	**2.15 (1.68–2.75)**	**<0.001**
**Region**				
Central (Ref)	536/1072 (50.0%)	1.00	1.00	–
North	164/364 (45.1%)	0.82 (0.64–1.05)	0.89 (0.68–1.17)	0.412
South	33/84 (39.3%)	0.65 (0.41–1.03)	0.71 (0.43–1.18)	0.189
**Gender**				
Male (Ref)	260/576 (45.1%)	1.00	1.00	–
Female	473/944 (50.1%)	1.22 (0.98–1.51)	1.08 (0.85–1.36)	0.524
**Experience**				
<2 years (Ref)	205/380 (53.9%)	1.00	1.00	–
2–10 years	418/836 (50.0%)	0.85 (0.66–1.10)	0.92 (0.69–1.22)	0.561
>10 years	110/304 (36.2%)	0.48 (0.35–0.66)	**0.65 (0.45–0.93)**	**0.019**
**Education**				
BSc (Ref)	479/1064 (45.0%)	1.00	1.00	–
PharmD	210/380 (55.3%)	1.51 (1.19–1.92)	**1.38 (1.08–1.77)**	**0.011**
MSc	52/76 (68.4%)	2.44 (1.38–4.32)	**2.10 (1.15–3.85)**	**0.016**
**Workload**				
Low (Ref)	182/304 (59.9%)	1.00	1.00	–
Medium	380/760 (50.0%)	0.67 (0.51–0.88)	0.78 (0.58–1.05)	0.102
High	171/456 (37.5%)	0.40 (0.29–0.55)	**0.55 (0.39–0.78)**	**0.001**

* Adjusted for all variables listed in the table using Multivariable Logistic Regression. Reference Category (Ref) represents an Odds Ratio of 1.00. Significant values *p* < 0.05 are highlighted in bold. Model Statistics: R^2^ = 0.312; Goodness-of-Fit Test: χ^2^ = 8.452, df = 8, *p* = 0.391.

## Data Availability

The original contributions presented in this study are included in the article and Supplementary Material. Further inquiries can be directed to the corresponding author.

## Supplementary Materials

The following supporting information can be downloaded at https://www.mdpi.com/article/10.3390/pharmacy14010031/s1, Table S1. Pearson Correlation Matrix of Independent Variables.
