# Supplementary material for: From Cosmetic Abuse to Clinical Mismanagement: A National Simulated Patient Study Assessing Community Pharmacists’ Stewardship of Topical Corticosteroids in Jordan
Source: Pharmacy (Basel). 2026 Feb 9;14(1):31. doi: 10.3390/pharmacy14010031 (PMC12921794; doi:10.3390/pharmacy14010031)
Supplement: Supplementary file 1 [file pharmacy-14-00031-s001.zip › pharmacy-4113571-supplementary.pdf]

Table S1. Pearson Correlation Matrix of Independent Variables

Variable	1	2	3	4	5	6
**1. Pharmacy Type (Chain)**	1.00					
**2. Region (Central)**	0.12	1.00				
**3. Gender (Female)**	0.05	-0.02	1.00			
**4. Experience (>10y)**	-0.15	0.08	-0.21*	1.00		
**5. Education (Postgrad)**	0.22*	0.04	0.09	0.11	1.00	
**6. Workload (High)**	0.31*	0.15	-0.05	-0.10	0.08	1.00

** Correlation is significant at the 0.05 level (2-tailed).*
